# Prickly Connections: Sociodemographic Factors Shaping Attitudes, Perception and Biological Knowledge about the European Hedgehog

**DOI:** 10.3390/ani13233610

**Published:** 2023-11-22

**Authors:** Ângela M. Ribeiro, Micaela Rodrigues, Nuno V. Brito, Teresa Letra Mateus

**Affiliations:** 1 CISAS—Center for Research and Development in Agrifood Systems and Sustainability, Polytechnic Institute of Viana do Castelo, NUTRIR (Technological Center for AgriFood Sustainability), Monte de Prado, 4960-320 Melgaço, Portugal; nunobrito@esa.ipvc.pt; 2 Escola Superior Agrária, Polytechnic Institute of Viana do Castelo, Rua Escola Industrial e Comercial de Nun’Àlvares, 4900-347 Viana do Castelo, Portugal; mrmicarodrigues7@gmail.com; 3 1H-TOXRUN—One Health Toxicology Research Unit, University Institute of Health Sciences, Cooperativa de Ensino Superior Politécnico e Universitário, 4585-116 Gandra, Portugal; 4 CECAV-Animal and Veterinary Research Centre, Associate Laboratory for Animal and Veterinary Sciences (AL4AnimalS), University of Trás-os-Montes and Alto Douro, Quinta de Prados, 5000-801 Vila Real, Portugal; 5 EpiUnit-Instituto de Saúde Pública da Universidade do Porto, Laboratório Para a Investigação Integrativa e Translacional em Saúde Populacional (ITR), Universidade do Porto, Rua das Taipas, nº 135, 4050-091 Porto, Portugal

**Keywords:** awareness, biodiversity stewardship, common knowledge, *Erinaceus europaeus*, human–nature interactions, perception, Portugal, questionnaire

## Abstract

**Simple Summary:**

The modern lifestyle, including indoor-centric living, urbanization and limited exposure to nature, contributes to the estrangement of humans from nature and a rapid decline in people’s natural history knowledge. Meanwhile, several wild species are adapting and thriving in urban environments alongside humans. How should we see the rising human disconnect with nature, even while urban wildlife increases and environmental education programmes are deployed? How does this lack of connection affect perception and attitude towards wildlife? What is the role of the sociodemographic context? To address these questions, we used a keystone species as a study model—the European hedgehog. We collected data via online questionnaires that comprised four main sections: (i) socio-demographic features; (ii) feelings, attitude and perception; (iii) natural history knowledge about species; and (iv) self-evaluation about the extent of knowledge and past experience. The data indicate generally positive feelings and attitudes towards hedgehogs. We found that academic qualifications and past experience with the species shaped people’s attitudes and natural history knowledge; however, the extent of knowledge, overall, was low and the study population was self-aware of this. We discuss the relevance of citizen profiling and possible avenues to enhance nature experience, improve knowledge, and increase public support for conservation measures.

**Abstract:**

The modern lifestyle of humans is leading to a limited exposure to nature. While several wild species are adapting and thriving in anthropic environments, natural history knowledge is declining, and positive attitudes and behaviours towards nature are facing challenges. Because anticipating attitudes and engendering broad-based support for nature-related measures requires a good grasp of social contexts, we set out to evaluate the sociodemographic factors driving the perception, attitudes towards, and natural history knowledge of a keystone species—the European hedgehog. In 2022, we conducted a questionnaire answered by 324 Portuguese adults. We found generally positive feelings and attitudes towards this species. A higher degree of academic qualifications and previous personal experience with the species seem to play a role in (i) people’s perception about human impacts on hedgehogs and (ii) positive attitudes, especially during encounters where the animals were in difficulty. Despite this, the extent of natural history knowledge was low overall, and the study population was self-aware of this. Our insights underline the need to tailor educational programmes if we are to encourage people to re-establish meaningful connections with nature, to foster social support for biodiversity stewardship, and to implement the One Health approach in a way that resonates with distinct social groups.

## 1. Introduction

“We are human in good part because of the particular way we affiliate with other organisms”.

E. O. Wilson. 1984. Biophilia—the human bond with other species, page 139.

Our experience of nature is declining. Regular interactions with nature have been progressively diminishing due to growing urbanization, indoor-centric living, sedentary lifestyles, and technological distractions. The consequence of this is an extinction of experience [1,2,3]. Despite the hardwired biophilic responses of humans to nature [4,5], the recognition of the positive effects of human–nature interactions on human health and wellbeing [6,7], and the fundamental role of these interactions for the future of ecosystems and biodiversity [8,9], there is mounting evidence pointing to the diminishing connection between people and nature [9].

As people become disconnected from the natural world, their familiarity with the natural environment, as assessed through their level of natural history knowledge (features of wildlife/ecosystems), declines. Therefore, there is a sense of detachment, little appreciation of the natural world and, ultimately, a lessening of positive attitudes and behaviours towards nature [3]. The consequences of experience extinction and the rapid decline in people’s natural history knowledge might be particularly challenging for wildlife conservation, as well as for the One Health framework. Traditionally, the approach to negative attitudes and behaviours is reinforcing conservation and health strategies with ecological knowledge. However, support has increased for the explicit incorporation of social dimensions, namely factors such as age, gender, level of education, urban vs. rural area of residence, and people’s perceptions [10,11]. Understanding stakeholders’ social contexts and perceptions makes it possible to anticipate the attitudes and behaviour [12,13,14] necessary for implementing design strategies that generate engagement and consequently ensure conservation and the success of public health programmes [15,16,17].

While the gap between people and nature is widening mostly due to the urbanization of human life, many wildlife species are colonizing and thriving in urban areas [18,19,20]. This is an unprecedented paradox: urban wildlife is increasing, efforts to raise awareness through public outreach campaigns are on the rise, and yet humans have less direct contact with nature than ever before. To explore this paradigm and its underlying drivers, we use the European hedgehog (*Erinaceus europaeus*, hereafter hedgehog) as a model species. The hedgehog, a ground-dwelling nocturnal mammal that is widespread across Europe in rural and urban habitats [21], was selected due to conservation concerns and its synanthropic behaviour (propensity to live in anthropic environments). Although the International Union for the Conservation of Nature (IUCN; [21]) and Red Book of Vertebrates of Portugal [22] list the species status as “least concern”, recent trends indicate that populations are plummeting in rural areas, while high densities near urbanized/humanized areas are increasing (e.g., [23,24,25]). Several factors contribute to these observations: exposure to pesticides and rodenticides in agricultural areas [26]; traffic collisions and mortality affecting dispersion and population dynamics [27]; and the decreased risk of predation in villages [28]. Regarding synanthropic behaviour, compelling studies demonstrate that hedgehogs are ecosystem sentinels for heavy metal(loid) pollution [29] and human health threats associated with zoonotic diseases [30,31]. Both hedgehogs’ ecology and synanthropic behaviour make this mammal a keystone species for agroecosystems and a sentinel for ecosystems and human health (One Health framework).

The concomitant evidence that estrangement from nature is increasing, interactions with natural world are enhancing emotional ties and positive attitudes, and European agroecosystems deserve conservation efforts [32] makes it timely and necessary to evaluate perception and natural history knowledge of a sentinel and keystone species. For this study, we set out to (i) assess citizens’ natural history knowledge about the hedgehog and (ii) evaluate the sociodemographic factors driving citizens’ perception and attitudes towards a common species whose populational trends have been affected, in some instances severely, by anthropogenic disturbances.

## 2. Materials and Methods

### 2.1. Data Collection: Questionnaire Design and Survey

The questionnaire design was based on a two-step approach: first, based on a bibliographic review of the topic, we designed a draft questionnaire that was tested with 15 respondents. After tailoring the questions, the final survey occurred in summer 2022 (July–August) using Google Forms and was rolled out following a “snowball” approach [33]. The “virtual snowball” sampling survey was disseminated via email through the mailing lists of researchers; these participants (primary respondents) were asked to disseminate the questionnaire with at least one of their personal contacts to proceed with the snowball and reach secondary respondents. All respondents were age > 18 and residents in Portugal. A small portion of respondents (seniors) requested a verbal survey. The final questionnaire included 35 questions organized into four main sections: the first block of questions collected information about respondents’ sociodemographic features; the second section gathered information on the respondents’ feelings, attitudes and perceptions towards the study species; the third part targeted respondents’ natural history knowledge of the hedgehog; and finally, in the fourth block, we assessed respondents’ self-evaluations about the species’ biology and their past experience with the study mammal (see Table S1). We adhered to Likert-type/scale questions, with a 6-point scale in which 1 is very negative/unimportant and 6 is very positive/important. The full questionnaire is provided as Supplementary Materials.

While conducting the questionnaires, we adhered to ethical principles as follows: (i) full disclosure—the respondents were fully informed about the scope and goal of the research; (ii) prior informed voluntary consent—consent was verbally/tacitly obtained from each respondent before conducting the questionnaire; and (iii) confidentiality—we ensured anonymity and privacy of the respondents.

### 2.2. Feelings, Attitudes Towards, and Perceptions about Hedgehogs

First, participants were questioned about their feelings towards the species (negative to positive on a scale from 1 to 6; Table S1). Next, we asked about their (i) attitudes towards a potential encounter with a distressed hedgehog, (ii) perceptions relating to the human impacts on the species mortality, (iii) perceptions of the need for management/conservation measures, and (iv) perceptions regarding the species’ impact on agriculture.

### 2.3. Biological Knowledge about Hedgehogs

To summarize the correctness of citizen natural history knowledge about hedgehog we used the information gathered in 19 questions to derive the *Erinaceus* Biological Knowledge Index (EBKI). The index was estimated as EBKI (1-i) = n° of correct answers/total n° of questions, where i is the total number of respondents; it is a continuous variable ranging from 0 to 1, where 1 indicates that all questions had a correct answer and 0 reflects completely incorrect answers.

### 2.4. Self-Evaluation and Past Experience with the Species

We asked the respondents to self-evaluate their knowledge about the species’ natural history (from “very poor” to “very good”), and subsequently inquired about whether they have ever seen a hedgehog alive in the wild, in a zoo/wildlife rescue centre, or in the media (Table S1).

### 2.5. Predictors of Attitudes, Perceptions, and Natural History Knowledge about Hedgehog

*Sociodemographic features*. We gathered sociodemographic information about the participants, such as age, gender, academic qualification, and profession/occupation. Afterwards, based on the professional activity/occupation reported (following the formal Portuguese classification of professions), we defined three social groups according to the potential to encounter/interact with/require information about hedgehogs in their professional/daily activity: 1—farmers (*n* = 18), 2—veterinary assistants, nurses and doctors and biologists (*n* = 31), and 3—others (*n* = 240).

*Urban–rural classification*. To characterize the level of urbanization of the participant’s area of residence, we used the Portuguese classification of urban areas; each parish of residence was assigned to one of three possible categories (Table S1).

### 2.6. Data Analysis

Descriptive statistics were used to summarize information about the study population. We used non-parametric tests as the data did not comply with normality as assessed by quantile–quantile plots and Tukey’s test. Kruskal–Wallis rank tests (χ^2^ value reported) were used to evaluate whether sociodemographic features, the level of urbanization of the residency area, and past experience impacted attitudes and perceptions. We tested (Student’s *t*-test) the hypothesis that the observed mean EBKI is the same as expected when assuming a normal distribution centred on 0.5 (sufficient knowledge). Next, because we were interested in understanding the effect of participants’ occupation on the level of EBKI, we performed a Kruskal–Wallis test followed by Dunn’s test for multiple pairwise comparisons. Finally, to predict whether the level of natural history knowledge about hedgehogs (EBKI) was explained by sociodemographic features and/or past experience of respondents, we used a partitioning approach through a regression tree, as implemented in rpart R package [34] and rpart.plot [35]. In brief, the tree was built by recursively identifying variables that cluster the dataset into two groups (“branches”), while minimizing the dissimilarity at the terminal nodes, according to the Gini criterion [36]. The partition ceases when no additional variables achieve further reductions in node impurity, as per the Gini criterion. To optimize the predictive performance, the trees were pruned to achieve minimal expected error and a 10-fold cross validation was implemented. All statistical analyses were performed using R (version 4.2.3) and R Studio (version 2022.02.3+492) with the packages gghalves, ggplot2, and ggstatsplot; *p*-values < 0.05 were considered statistically significant.

## 3. Results

### 3.1. Socio-Demographic Features of Respondents

We had 324 participants in our survey aged between 18 and 93 years old (237 females, 86 males, and 1 non-binary participant) and living in 82 Portuguese municipalities (from north to south). Most of the sample corresponded to young adults (66.7%; 18–44 years old) and 14.5% were seniors (>65 years old). While 76.5% of the respondents lived in urban areas, 3.4% resided in rural parishes. More than half of the sample (60.5%) reported having higher academic qualifications (honours/licentiate, master’s or doctoral degrees) and a reduced number (2.5%) were illiterate. The respondents more likely to deal with hedgehogs due to their profession/occupation (i.e., farmers (*n* = 18), biologists (*n* = 5), veterinary assistants/nurses/doctors (*n* = 26)) represented 23.8% of the sample. This differential participation is noteworthy and thus discussed later.

### 3.2. Feeling, Attitude and Perception Regarding Hedgehogs

Many respondents revealed mostly positive feelings about the species (83.3%, scores 4, 5 and 6); only 16.7% reported mostly negative feelings (scores 1, 2 and 3). Attitudes towards hedgehogs was, in general, positive. The most popular attitude towards a likely encounter with a hedgehog in difficulty was to “seek help” (74.4%), either by contacting the authorities or a wildlife rescue centre; on the other side of the scale, 1.4% reported an attitude of killing the animal. Ignoring the situation was the attitude supported by 12.8% of the respondents.

Less than half of the participants (42.9%) perceive humans as a factor of high impact on hedgehog mortality, whereas 6.6% agreed that human impacts are “very unimportant” (Table S1). There was a general consensus for the need to protect the species (90.4%), but some participants thought that it needed to be controlled (7.7%) or eliminated (1.9%). The impact of the species on farming was unevenly appreciated: 36.1% of the participants were worried about the negative impacts (scores 1, 2, and 3; Table S1), whereas 63.9% recognized the benefits of hedgehogs in agriculture (scores 4, 5, and 6; Table S1).

### 3.3. Common Knowledge about Hedgehog Biology and Conservation

**Figure 1:**
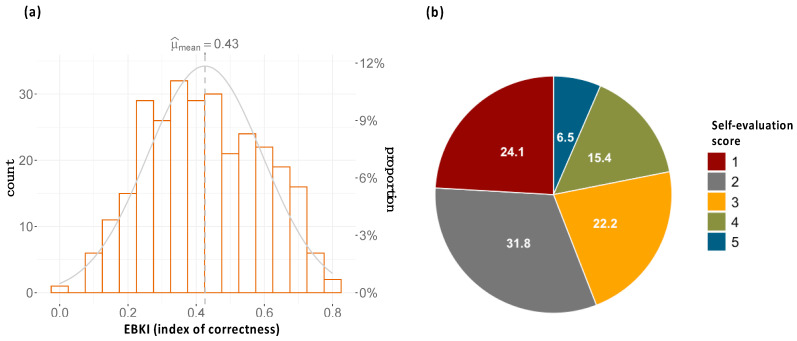
Assessment of common knowledge about hedgehog’s biology. (**a**) Distribution of citizen knowledge correctness estimated as EBKI (ranges: 0–1). μ = observed mean value for the index estimated based on 289 respondents. Student’s t-test revealed that EBKI was significantly lower than the value expected, assuming a normal distribution centred around 0.5 (sufficient knowledge). (**b**) Respondents’ knowledge self-evaluation. The scores provided in the questionnaire ranged from 1 to 6, yet category 6 “very good” had zero observations.

Only 289 respondents fully answered the 19 questions regarding the natural history of the species, and 64.1% showed a low level of knowledge (EBKI < 0.5; Figure 1a). The knowledge about hedgehogs’ natural history (mean EBKI = 0.43) was significantly lower than expected if assuming a population with normal distribution cantered on 0.5 (t(288) = −7.46, *p* < 0.001). A close inspection of the results revealed the following: most respondents (91.7%) recognized hedgehogs as a rural dweller species; in several questions, participants reported “do not know”, with the highest frequency for two questions inquiring about breeding biology (for litter number per year and number of offspring, 68.8% and 46.6% reported a lack of knowledge, respectively).

### 3.4. Self-Knowledge and Past Experience

In general, the respondents self-evaluated their knowledge about hedgehogs’ biology as quite poor (scores 1 and 2 = 55.9%; Figure 1b). For 9.0% of the respondents, the hedgehog has become a species primarily accessed through the filters of media (i.e., books, magazines, TV, or online) and 7.6% had never had an encounter with the species; however, most participants (83.4%) reported observations in the wild or in captive conditions (zoos or rescue centres).

### 3.5. Citizen Profile as a Driver of Attitudes, Perceptions and Common Knowledge

Our results show that attitude or perception is neither affected by gender nor the level of urbanization in the parish of residency of participants (Table 1). In contrast, the academic qualification of the respondents is related to the attitude and the three assessments of perception (all χ^2^ tests of independence are significant; Table 1). Attitude and perception are associated with occupation/profession, except for the perceived impact that hedgehogs have on farming (Table 1). Upon closer examination, the results reveal that, generally, the proportions inside each explanatory category of perception were not equal and thus significant (see portions test in Figures S1–S5). For instance, the perceptions that humans have no impact on hedgehog mortality, that the species needs culling, and that hedgehogs have a negative impact on farming were mostly conveyed by participants with basic levels of education (academic qualification: first cycle). Regarding past experiences and encounters, i.e., type of previous observation of the species, it was also related to attitudes towards a hedgehog in difficulty, the perception of hedgehogs’ impact on farming, and the perception of the measures of conservation/management, but did not relate to the perception that humans influence species mortality (Table 1).

**Figure 2:**
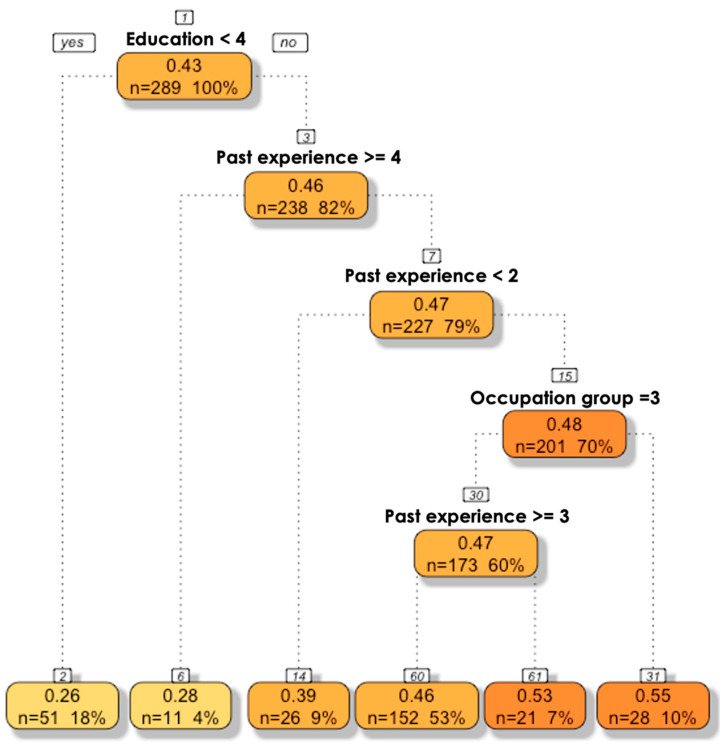
Decision tree to predict EBKI. *Academic qualification* (Ac. Qualif.) is self-explanatory (see Table S1 for levels). *Occupation/profession* pertains the social functional group used as a proxy for participants’ likelihood of encountering/interacting with hedgehogs in their professional or daily activities: 1: farmers, 2: biologist and veterinary doctors/nurses/assistant, 3: others. *Past experience* refers to the participants past observation of a hedgehog either in real life or through the media, or never seen it. Reading the tree: the first split, at the root node, asked “at mean EBKI (0.43), is the academic qualification of the participant in basic levels (illiterate + 1st cycle + 2nd cycle)”? Negative or “no” responses branched to the right. If “no” (negative responses branch to the right), the second question inquired whether “the respondent had never seen a hedgehog” or if “yes” (left branch) only via media. In participants with higher levels of academic qualification who have seen the species before, a third question followed: “does the respondent belong to the occupation group less likely to encounter a hedgehog in their professional or occupational activities?”, if “no” participants were classified as the ones with the largest EBKI (0.55; 10% of the sampled population).

**Figure 3:**
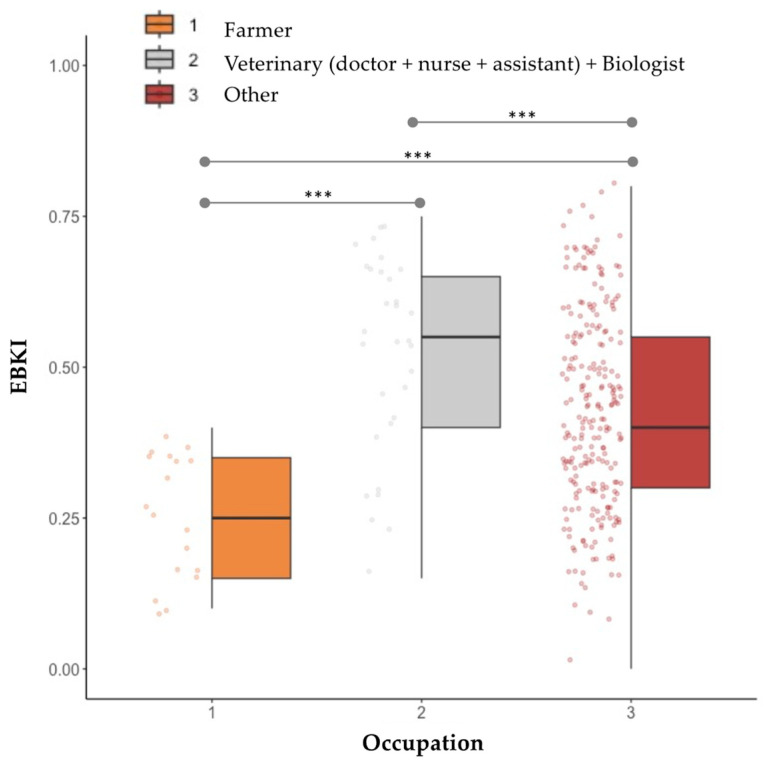
Effect of occupation on EBKI. Testing the role of participant’s occupation on the correctness of knowledge about species biology; the three groups were defined according to their likelihood of encountering/interacting with a hedgehog during their professional/daily activities. Horizontal lines connecting boxplots indicate pairwise comparisons (1–2; 1–3, 2–3) and *** indicates *p* < 0.001.

Most participants knew that hedgehogs are solitary mammals (51.6%; 26.6% reported no knowledge) that hibernate (72.3%; 21.1% indicated no knowledge) and were confident that it is mostly a rural-dwelling species (91.7%). Some participants (5.0%) expressed that keeping a hedgehog as a pet animal is a legal practice (21.5% reported an absence of knowledge). The regression tree analysis for predicting the factors shaping EBKI included five splits with six leaf nodes and three variables (Figure 2). The provided model included the five explanatory variables (Table 1), but only three are needed to explain the variation in the dataset. The first split in the decision tree is associated with academic qualification and explains 21.5% of the variance in the data; the second split relates to past experience and helps to explain another 4.8% of variance; and the fourth split accounts for the effect of occupation. Higher levels of academic qualification, seeing the species live (either in the wild or captive) and belonging to an occupation group other than “farmers” are the predictors for largest EBKI (0.55). Further exploring the occupation/profession effect, we found significant differences in EBKI among the three functional groups (Kruskal–Wallis χ^2^ = 28.89, *p* < 0.001), with “farmers” presenting the lowest index of correctness (Figure 3).

## 4. Discussion

The Western European hedgehog is a cosmopolitan mammal found in the countryside, as well as being common in suburban and urban areas. In Portugal, it is a popular animal, which (i) evokes positive feelings, (ii) generally receives positive attitudes, (iii) prompts positive perception regarding the needs for conservation, and (iv) leads to high levels of awareness regarding the negative impact that humans have on the species. The general positive attitudes and feelings towards hedgehogs did not equate to natural history knowledge: the lack of knowledge was evident by the mean EBKI = 0.43 and by the high frequency of replies of “do not know”. The participants were self-aware about their limited knowledge and surprisingly honest about it.

### 4.1. Citizen Profile

The social perception and natural history knowledge about hedgehogs are basic issues related to education and occupation/profession. There is a discrepancy in both perception and EBKI between participants with lower degrees of qualification (i.e., illiterate, first, second) vs. people with high school or university qualifications. Respondents with lower academic qualifications (all age > 65) perceive hedgehogs as detrimental for agriculture and in need of culling, and they believe that their decreasing populational trend is not affected by humans. Contrastingly, participants with higher academic qualifications mostly reported positive attitudes and perceptions. This pattern may reflect (i) farmers’ general intolerance and negative attitudes towards wildlife due to potential agricultural damages or simply due to social/cultural norms, as noted by Jordan et al. [37], as well as (ii) the awareness of wildlife conservation and environmental issues taught in high schools and universities. The apparent generational shift may result from wider societal experiences or shifting cultural norms. It is widely known that the way individuals observe, understand, interpret, and evaluate a given object/experience/outcome (i.e., perception) and the culmination of feelings or opinions regarding that same issue (i.e., attitude) is shaped by several personal factors, as well as cultural norms and beliefs [10]. Considering the social context in a given ecological system can provide insights to create opportunities to reconnect with nature, design awareness-raising programmes, and avoid polarized opinions that perpetuate human–wildlife conflicts.

### 4.2. Opportunities for Reconnecting with Nature—Education

Resolving the lack of experience of nature requires opportunities for meaningful interactions with the natural world [2]. Although 76% of the citizens reported an encounter with a wild hedgehog and nearly half of the participants reported positive feelings about the species, the low level of natural history knowledge is striking and indicates the pressing need to implement education programmes. For instance, in Portugal, a country where hedgehogs are identified as reservoirs of zoonotic diseases [38,39], half of the respondents (53%) reported no knowledge about this issue, and a quarter stated that hedgehogs do not transmit any diseases, either to humans or other animals. Furthermore, the lack of knowledge of simple biological facts, such as habitat preferences (cosmopolitan), social behaviour (solitary mammal), seasonal dormancy (hibernation), and the illegality of keeping a captive animal, call for urgent action.

The general public still perceives hedgehogs as a rural mammal, when several studies have highlighted their increasing presence, sometimes reaching high densities, in human environments [20,39]. Their synanthropy increases the likelihood and frequency of contact with pathogens from domestic animals and humans, increasing the potential for zoonotic transmission [25,31]. Given the above, we not only need to revert the alienation from nature by providing opportunities to experience nature [14], but also need to deploy bold educational policy changes that shift from “one fits all” paradigm. We require macro (European) and meso (national) One Health and biodiversity conservation educational and outreach programmes to be tailored to the micro scale, i.e., to meet the regional/local community features (perception, attitudes, knowledge).

### 4.3. Implications for Conservation Strategies and Eco-Schemes

The hedgehog is a key indicator of a healthy and sustainable farmland (arable land and pastures), so its absence must be a serious concern for agriculture. In fact, along with urban expansion and traffic accidents, farming intensification is pointed out as one of the main factors threating hedgehog populations [24,26,39]. Notwithstanding, the EU Common Agricultural Policy eco-schemes (designation under CAP 2023-2027; previously agri-environment schemes) have no direct measures for targeting this keystone species. Our results, which indicate a public perception that hedgehogs have positive impacts on farming despite an evident lack of natural history knowledge, highlight the need for national and regional authorities to implement CAP to incorporate farmland management measures that benefit hedgehogs [39]. The negative perceptions and smaller EBKI of Portuguese farmers should grant further research to assess farmers willingness to adopt eco-schemes dedicated to hedgehogs, so the national authorities can tailor effective conservation strategies [40,41]. It is evident that only a good grasp of the local social context of human–nature interactions will allow the implementation of widely accepted biodiversity conservation plans [9,10,42].

### 4.4. Study Caveats

The survey was distributed in two formats: online and face-to-face (for elderly and mostly illiterate). We were aware of the bias it could introduce, hence we read the questions to the participants very carefully, did not express any opinions ourselves, and ensured that we had their verbal informed consent. Moreover, we contend that our survey strategy may have caused some misrepresentation of groups, which might have partially affected the conclusions. For instance, males were under-represented (females: 73.5% vs. males: 22.8%) when considering the Portuguese population as reference (females: 52.4% vs. males: 47.6%); however, this gender difference in response rates is not exclusive to our study; in fact, it is well-known and widely discussed [43]. Additionally, there was a lower representation of seniors (14.5%, age > 65) when the recent reference demographic parameter is 23.4% [44]. Therefore, we refrained from further considerations regarding gender and age. Despite an apparent small sample size for farmers in the profession/occupational groups (6.2%), it fits well the Portuguese population with full-time employment in agriculture (<5%) [45]. Notably, the farmer participants were mostly seniors and had the lowest academic qualifications in the sample (illiterate or first cycle). Hence, once again, we are cautious with the interpretation of our results.

## 5. Conclusions

It is important to recognize that attitudes towards nature are not static and can be influenced and shaped over time through targeted efforts. Although direct experiences with nature foster a sense of appreciation and empathy and deem the natural world fundamental to people’s lives, they may not be sufficient. In light of our findings, we advocate for the creation of strategies to identify and engage with local and relevant stakeholders. For instance, it is crucial that the sector with the largest power helps to reverse the current declining trends of rural hedgehog populations, i.e., encourage the farming sector to get involved with the development of integrated and sustainable management of the rural landscape. We suggest addressing the gap between the growth of scientific knowledge production and farming practices via the new eco-scheme policy instruments. Likewise, we call for the inclusion of social groups’ perceptions when tailoring educational programmes pertaining One Health topics, particularly with regard to wild species that are becoming urban dwellers. Although synanthropy can bring benefits, such as increasing biodiversity in urban areas and improving human physical and mental health, there is also a risk of exposure to pathogens due to extensive lack of knowledge. We strongly believe that studying citizen profiles and social groups is crucial for the development of strategies that account for the diverse ways in which humans, animals, and the environment interact, ultimately leading to improved health outcomes for all, as desired in a One Health approach. Raising awareness towards conservation needs and deploying interventions targeting specific players are crucial for hedgehog conservation.

## Figures and Tables

**Table 1:** Summary of the Chi-square tests of independence for attitudes and perceptions from sociodemographic factors. Dependent and explanatory variables are all categorial (details in Table S1).

	Attitude		Perception	
Explanatory	Towards a Hedgehog in Difficulties	Management/Conservation Measures	Human Effect on Mortality	Hedgehog Impact on Farming
Gender	χ^2^ = 10.19 *p* = 0.25 VCramer = 0.06	χ^2^ = 0.49 *p* = 0.97 VCramer = 0.00	χ^2^ = 4.38 *p* = 0.93 VCramer = 0.00	χ^2^ = 6.85 *p* = 0.74 VCramer = 0.00
Level of urbanization	χ^2^ = 5.77 *p* = 0.67 VCramer = 0.00	χ^2^ = 2.26 *p* = 0.69 VCramer = 0.00	χ^2^ = 10.94 *p* = 0.36 VCramer = 0.04	χ^2^ = 6.51 *p* = 0.85 VCramer = 0.00
Academic qualification	χ^2^ = 171.56 *p* = 1.43 × 10^−22^ VCramer = 0.35	χ^2^ = 78.19 *p* = 6.12 × 10^−11^ VCramer = 0.33	χ^2^ = 153.08 *p* = 1.03 × 10^−18^ VCramer = 0.29	χ^2^ = 72.11 *p* = 2.23 × 10^−4^ VCramer = 0.16
Occupation	χ^2^ = 77.82 *p* = 1.34 × 10^−13^ VCramer = 0.35	χ^2^ = 41.95 *p* = 1.71 × 10^−8^ VCramer = 0.26	χ^2^ = 70.39 *p* = 3.72 × 10^−11^ VCramer = 0.32	χ^2^ = 14.47 *p* = 0.15 VCramer = 0.09
Past experience	χ^2^ = 43.20 *p* = 2.61 × 10^−4^ VCramer = 0.15	χ^2^ = 16.71 *p* = 0.03 VCramer = 0.12	χ^2^ = 25.40 *p* = 0.19 VCramer = 0.07	χ^2^ = 39.32 *p* = 6.08 × 10^−3^ VCramer = 0.13

VCramer [0.10–0.20] indicates a weak association; VCramer [0.20–0.40] indicates a moderate association.

## Data Availability

Anonymized datasets are available from the corresponding authors upon reasonable request.

## Supplementary Materials

The following supporting information can be downloaded at: https://www.mdpi.com/article/10.3390/ani13233610/s1. Supplementary Text (informed consent and questionnaire), Table S1: Details about the data collected. Variables used for the statistical analyses and their attributes. Figure S1: Proportions test for gender in each category (defined in Table S1) for three perception variables assessed; Figure S2: Proportions test for Academic Qualification in each category (defined in Table S1) for three perception variables assessed; Figure S3: Proportions test for Occupation in each category (defined in Table S1) for three perception variables assessed; Figure S4: Proportions test for Occupation in each category (defined in Table S1) for three perception variables assessed; Figure S5: Proportions test for the level of urbanization in the participants residency area—residency typology—for each category (defined in Table S1) for three perception variables assessed.

## References

[1] Pyle R.M. (1993). The Thunder Tree.

[2] Miller J.R. (2005). Biodiversity Conservation and the Extinction of Experience. Trends Ecol. Evol..

[3] Soga M., Gaston K.J. (2016). Extinction of Experience: The Loss of Human–Nature Interactions. Front. Ecol. Environ..

[4] Kellert S.R., Wilson E.O. (1993). The Biophilia Hypothesis.

[5] Wilson E.O. (1986). Biophilia.

[6] Hartig T., Mitchell R., de Vries S., Frumkin H. (2014). Nature and Health. Annu. Rev. Public Health.

[7] White M.P., Elliott L.R., Grellier J., Economou T., Bell S., Bratman G.N., Cirach M., Gascon M., Lima M.L., Lõhmus M. (2021). Associations between Green/Blue Spaces and Mental Health across 18 Countries. Sci. Rep..

[8] Otto S., Pensini P. (2017). Nature-Based Environmental Education of Children: Environmental Knowledge and Connectedness to Nature, Together, Are Related to Ecological Behaviour. Glob. Environ. Chang..

[9] Zaradic P.A., Pergams O.R.W., Kareiva P. (2009). The Impact of Nature Experience on Willingness to Support Conservation. PLoS ONE.

[10] Dickman A.J. (2010). Complexities of Conflict: The Importance of Considering Social Factors for Effectively Resolving Human–Wildlife Conflict. Anim. Conserv..

[11] Šūmane S., Kunda I., Knickel K., Strauss A., Tisenkopfs T., Rios I.d.I., Rivera M., Chebach T., Ashkenazy A. (2018). Local and Farmers’ Knowledge Matters! How Integrating Informal and Formal Knowledge Enhances Sustainable and Resilient Agriculture. J. Rural Stud..

[12] Castillo-Huitrón N.M., Naranjo E.J., Santos-Fita D., Estrada-Lugo E. (2020). The Importance of Human Emotions for Wildlife Conservation. Front. Psychol..

[13] Rosa C.D., Collado S. (2019). Experiences in Nature and Environmental Attitudes and Behaviors: Setting the Ground for Future Research. Front. Psychol..

[14] Soga M., Gaston K.J. (2020). The Ecology of Human–Nature Interactions. Proc. R. Soc. B.

[15] Straka T.M., Miller K.K., Jacobs M.H. (2020). Understanding the Acceptability of Wolf Management Actions: Roles of Cognition and Emotion. Hum. Dimens. Wildl..

[16] Ceríaco L.M., Marques M.P., Madeira N.C., Vila-Viçosa C.M., Mendes P. (2011). Folklore and Traditional Ecological Knowledge of Geckos in Southern Portugal: Implications for Conservation and Science. J. Ethnobiol. Ethnomedicine.

[17] Manfredo M.J. (2008). Who Cares About Wildlife?.

[18] Ives C.D., Lentini P.E., Threlfall C.G., Ikin K., Shanahan D.F., Garrard G.E., Bekessy S.A., Fuller R.A., Mumaw L., Rayner L. (2015). Cities Are Hotspots for Threatened Species: The Importance of Cities for Threatened Species. Glob. Ecol. Biogeogr..

[19] Alagona P.S. (2022). The Accidental Ecosystem: People and Wildlife in American Cities.

[20] Hubert P., Julliard R., Biagianti S., Poulle M.-L. (2011). Ecological Factors Driving the Higher Hedgehog (Erinaceus Europeaus) Density in an Urban Area Compared to the Adjacent Rural Area. Landsc. Urban Plan..

[21] Amori G. (2016). Erinaceus Europaeus. The IUCN Red List of Threatened Species.

[22] Mathias M.d.L., Fonseca C., Rodrigues L., Grilo C., Lopes-Fernandes M., Palmeirim J.M., Santos-Reis M., Alves P.C., Cabral J.A., Ferreira M., Fciências I.D. (2023). Livro Vermelho Dos Mamíferos de Portugal Continental.

[23] Hof A.R., Bright P.W. (2016). Quantifying the Long-Term Decline of the West European Hedgehog in England by Subsampling Citizen-Science Datasets. Eur. J. Wildl. Res..

[24] Pettett C.E., Johnson P.J., Moorhouse T.P., Macdonald D.W. (2018). National Predictors of Hedgehog Erinaceus Europaeus Distribution and Decline in Britain. Mammal Rev..

[25] Taucher A.L., Gloor S., Dietrich A., Geiger M., Hegglin D., Bontadina F. (2020). Decline in Distribution and Abundance: Urban Hedgehogs under Pressure. Animals.

[26] Dowding C.V., Shore R.F., Worgan A., Baker P.J., Harris S. (2010). Accumulation of Anticoagulant Rodenticides in a Non-Target Insectivore, the European Hedgehog (Erinaceus Europaeus). Environ. Pollut..

[27] Moore L.J., Petrovan S.O., Baker P.J., Bates A.J., Hicks H.L., Perkins S.E., Yarnell R.W. (2020). Impacts and Potential Mitigation of Road Mortality for Hedgehogs in Europe. Animals.

[28] Pettett C.E., Moorhouse T.P., Johnson P.J., Macdonald D.W. (2017). Factors Affecting Hedgehog (Erinaceus Europaeus) Attraction to Rural Villages in Arable Landscapes. Eur. J. Wildl. Res..

[29] Jota Baptista C., Seixas F., Gonzalo-Orden J.M., Patinha C., Pato P., Ferreira da Silva E., Casero M., Brazio E., Brandão R., Costa D. (2023). High Levels of Heavy Metal(loid)s Related to Biliary Hyperplasia in Hedgehogs (Erinaceus europaeus). Animals.

[30] Ruszkowski J.J., Hetman M., Turlewicz-Podbielska H., Pomorska-Mól M. (2021). Hedgehogs as a Potential Source of Zoonotic Pathogens-A Review and an Update of Knowledge. Animals.

[31] Jota Baptista C., Oliveira P.A., Gonzalo-Orden J.M., Seixas F. (2023). Do Urban Hedgehogs (Erinaceus europaeus) Represent a Relevant Source of Zoonotic Diseases?. Pathogens.

[32] Pe’er G., Finn J.A., Díaz M., Birkenstock M., Lakner S., Röder N., Kazakova Y., Šumrada T., Bezák P., Concepción E.D. (2022). How Can the European Common Agricultural Policy Help Halt Biodiversity Loss? Recommendations by over 300 Experts. Conserv. Lett..

[33] Aguiar A., Pinto M., Duarte R. (2021). Psychological Impact of the COVID-19 Pandemic and Social Determinants on the Portuguese Population: Protocol for a Web-Based Cross-Sectional Study. JMIR Res. Protoc..

[34] Therneau T., Atkinson B. (2022). _rpart: Recursive Partitioning and Regression Trees_. Rpackage version 4.1.19. https://CRAN.R-project.org/package=rpart.

[35] Milborrow S. (2022). _rpart.plot: Plot ‘rpart’ Models: An Enhanced Version of ‘plot.rpart’_. R package version 3.1.1. https://CRAN.R-project.org/package=rpart.plot.

[36] Breiman L. (2022). Classification And Regression Trees.

[37] Jordan N.R., Smith B.P., Appleby R.G., Eeden L.M., Webster H.S. (2020). Addressing Inequality and Intolerance in Human–Wildlife Coexistence. Conserv. Biol..

[38] Barradas P.F., Mesquita J.R., Mateus T.L., Ferreira P., Amorim I., Gärtner F., Sousa R. (2021). de Molecular Detection of Rickettsia Spp. in Ticks and Fleas Collected from Rescued Hedgehogs (Erinaceus Europaeus) in Portugal. Exp. Appl. Acarol..

[39] Yarnell R.W., Pettett C.E. (2020). Beneficial Land Management for Hedgehogs (Erinaceus Europaeus) in the United Kingdom. Animals.

[40] Kusmanoff A.M., Fidler F., Gordon A., Garrard G.E., Bekessy S.A. (2020). Five Lessons to Guide More Effective Biodiversity Conservation Message Framing. Conserv. Biol..

[41] Massfeller A., Meraner M., Hüttel S., Uehleke R. (2022). Farmers’ Acceptance of Results-Based Agri-Environmental Schemes: A German Perspective. Land Polic..

[42] Bennett N.J., Roth R., Klain S.C., Chan K., Christie P., Clark D.A., Cullman G., Curran D., Durbin T.J., Epstein G. (2017). Conservation Social Science: Understanding and Integrating Human Dimensions to Improve Conservation. Biol. Conserv..

[43] Tourangeau R., Rips L.J., Rasinski K. (2000). The Psychology of Survey Response.

[44] INE—National Statistics Institute https://censos.ine.pt.

[45] FFMS—Fundação Francisco Manuel dos Santos https://www.pordata.pt/en.

