# Supplementary material for: Prickly Connections: Sociodemographic Factors Shaping Attitudes, Perception and Biological Knowledge about the European Hedgehog
Source: Animals (Basel). 2023 Nov 22;13(23):3610. doi: 10.3390/ani13233610 (PMC10705511; doi:10.3390/ani13233610)
Supplement: Supplementary file 1 [file animals-13-03610-s001.zip › FigureS1-5.pdf]

Supplementary Figures S1 to S5 for "Prickly connections: sociodemographic factors shaping attitudes, perception and biological knowledge about hedgehog" by Ribeiro et al. 2023.

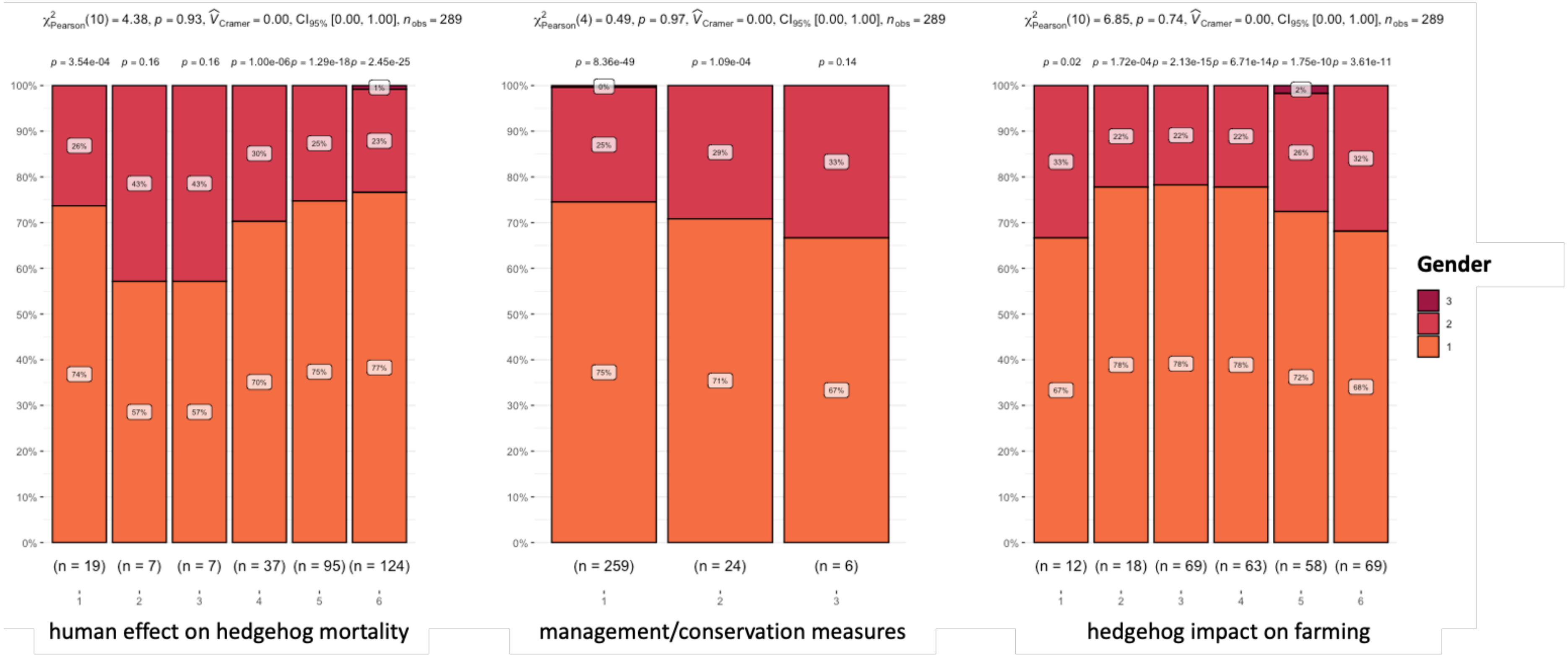

Figure S1. Proportions test for gender in each category (defined in TableS1) for three perception variables assessed: a) perceived human effect on hedgehog mortality, b) perceived need for management/conservation measures regarding hedgehogs; c) perceived impact of hedgehog on farming.

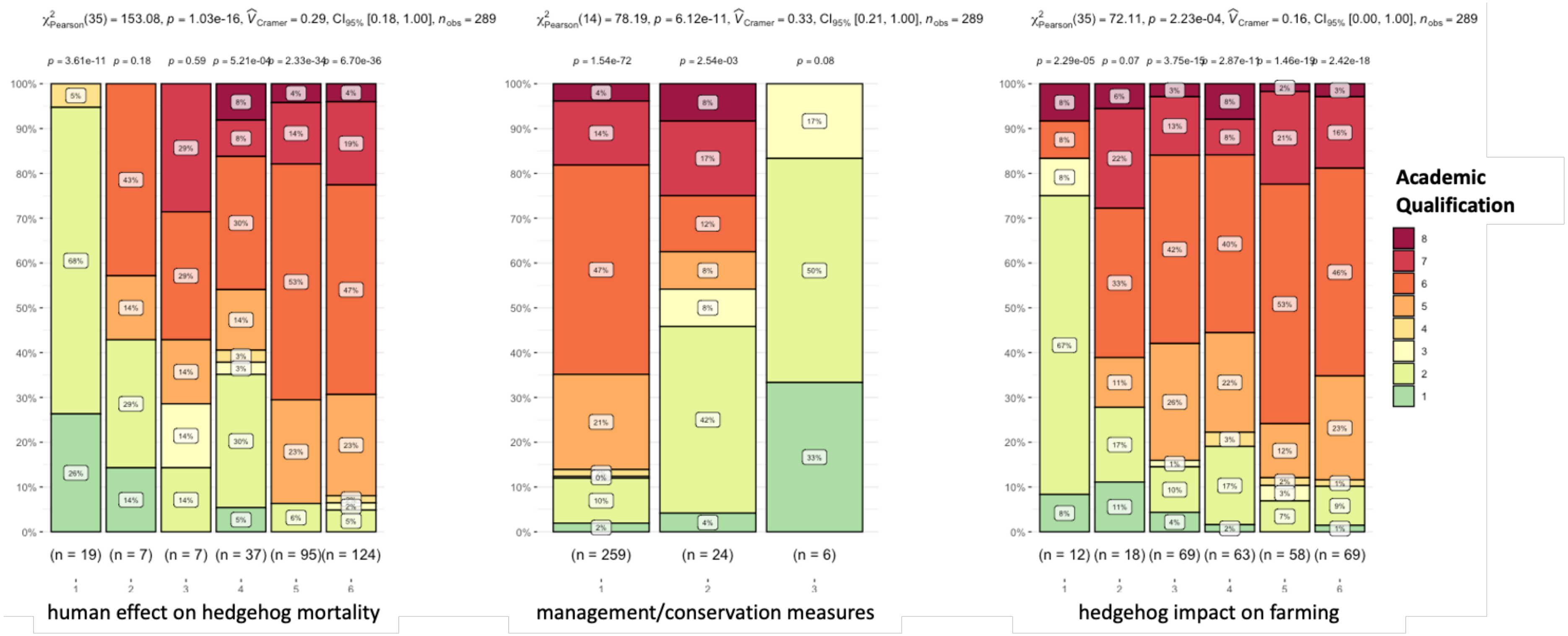

Figure S2. Proportions test for Academic Qualification in each category (defined in TableS1) for three perception variables assessed: a) perceived human effect on hedgehog mortality, b) perceived need for management/conservation measures regarding hedgehogs; c) perceived impact of hedgehog on farming.

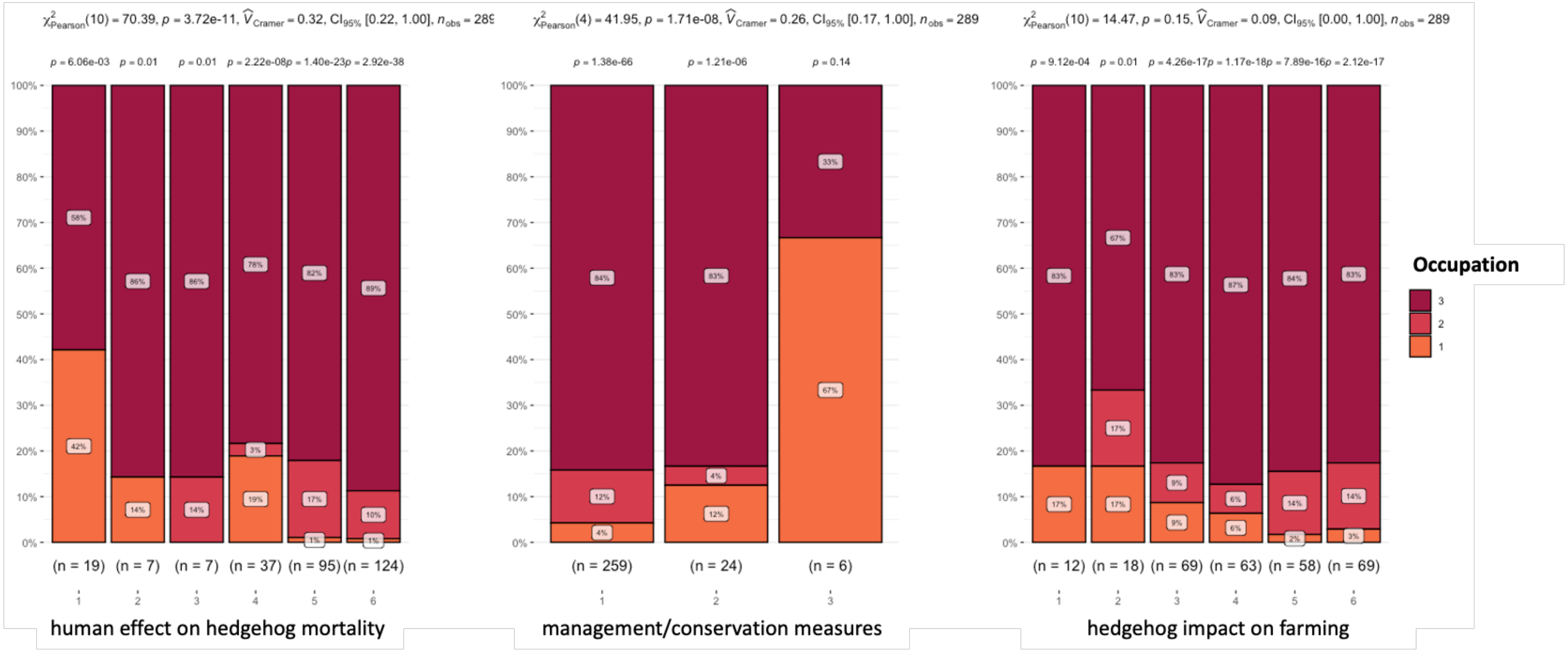

Figure S3. Proportions test for Occupation in each category (defined in TableS1) for three perception variables assessed: a) perceived human effect on hedgehog mortality, b) perceived need for management/conservation measures regarding hedgehogs; c) perceived impact of hedgehog on farming.

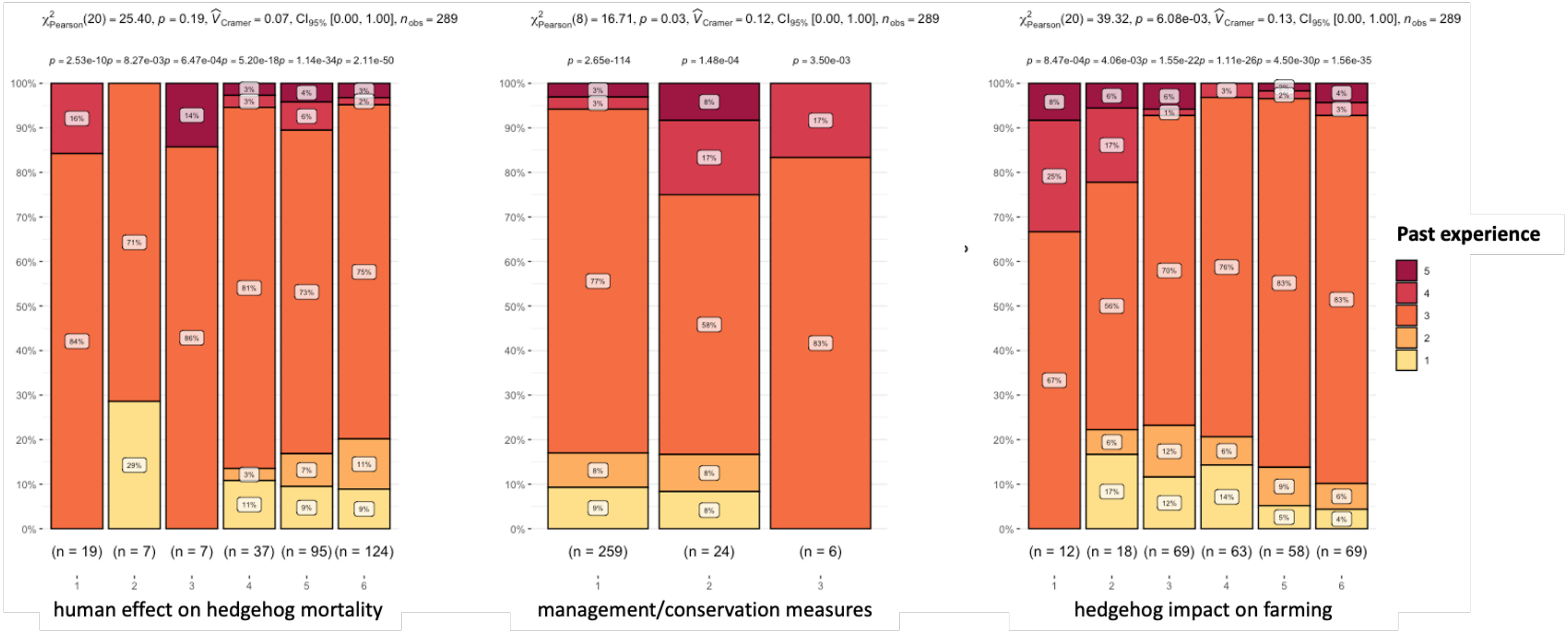

Figure S4. Proportions test for Occupation in each category (defined in TableS1) for three perception variables assessed: a) perceived human effect on hedgehog mortality, b) perceived need for management/conservation measures regarding hedgehogs; c) perceived impact of hedgehog on farming.

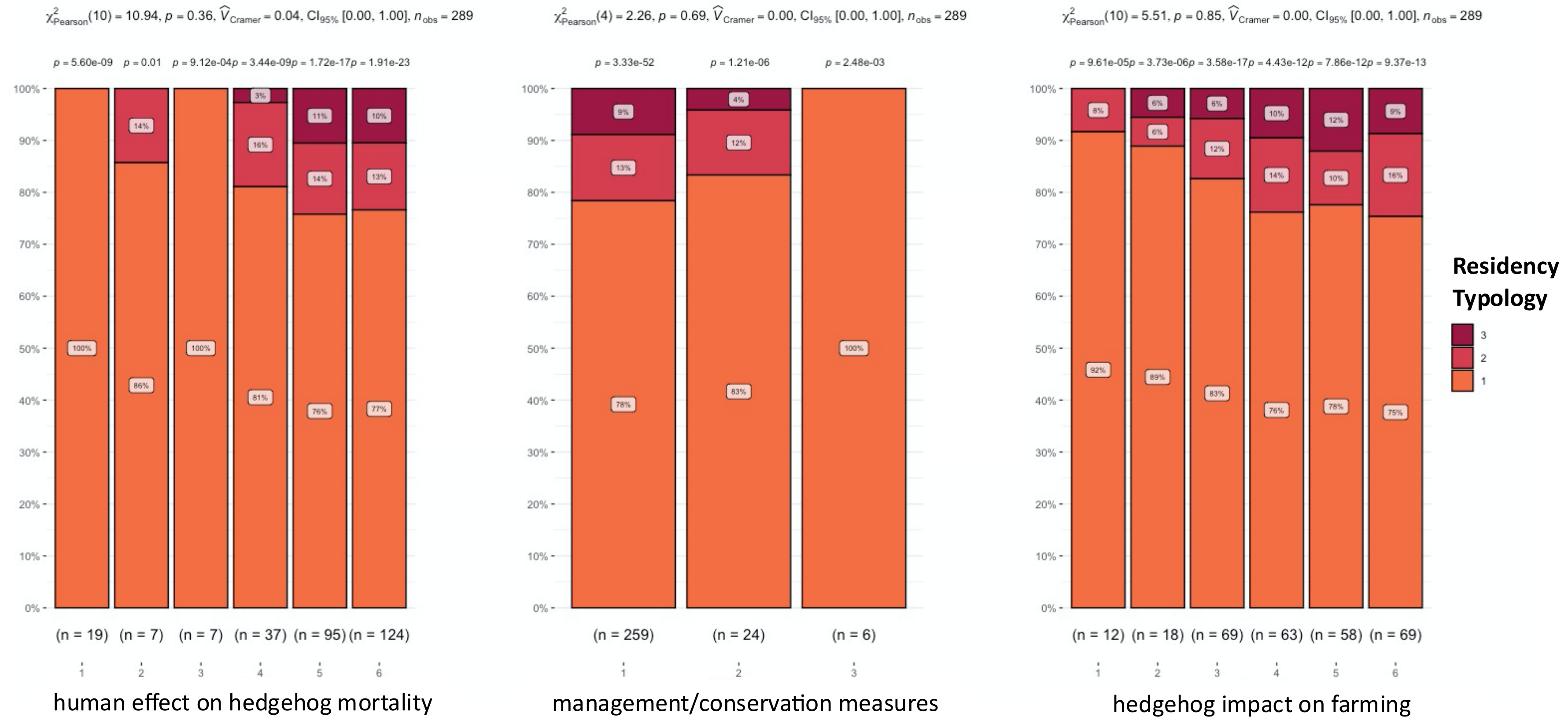

Figure S5. Proportions test for the level of urbanization in the participants residency area – residency typology - for each category (defined in TableS1) for three perception variables assessed: a) perceived human effect on hedgehog mortality, b) perceived need for management/conservation measures regarding hedgehogs; c) perceived impact of hedgehog on farming.
