# Supplementary material for: Prickly Connections: Sociodemographic Factors Shaping Attitudes, Perception and Biological Knowledge about the European Hedgehog
Source: Animals (Basel). 2023 Nov 22;13(23):3610. doi: 10.3390/ani13233610 (PMC10705511; doi:10.3390/ani13233610)
Supplement: Supplementary file 1 [file animals-13-03610-s001.zip › SuppText_ConsentQuestionnaire.pdf]

Supplementary text for "Prickly connections: sociodemographic factors shaping attitudes, perception and biological knowledge about hedgehog" by Ribeiro et al. 2023.

1. Informed consent – page1-2
2. Questionnaire (as presented in the *Google Forms*) – page 3-9

---

1. Informed consent

Please read the following information carefully. The predicted time to answer this questionnaire is approximately 9 minutes.

The study "Cross-sectional study on the sensitivity and knowledge of the adult population of continental Portugal about the European hedgehog" is part of the Veterinary Nursing degree programme at *Escola Superior Agrária do Instituto Politécnico de Viana do Castelo* (ESA-IPVC), Portugal.

The aim of this study is to assess the awareness and knowledge of the adult population (citizens over the age of 18) of continental Portugal about the European hedgehog. Therefore, the intent is to assess knowledge about the biology and diseases affecting this animal species, as well as the population's perception and attitudes towards the study species. To this end, an online questionnaire survey will be carried out using the *Google Forms* platform. Sociodemographic data will be collected from the participants (e.g., age, gender, place of residence. occupation) and questions will be asked in order to assess participant´s opinion and knowledge about this animal species. The survey will be disseminated via social media and via email to the researcher's contacts, and it will be requested their dissemination via the method known as snowballing. Only participants resident in continental Portugal and aged 18 or over will be included. The data obtained will be important for obtaining scientific knowledge on this subject, which will allow to create more efficient environmental education strategies about this species.

Participation is voluntary and does not involve any financial risks or benefits for the participants. Participation in the study is entirely voluntary and non-participation will not cause any harm; the respondent can leave the study at any time, which will not result in any harm. However, because participation in the study is anonymous, once the survey has been submitted, it will not be possible to withdraw, as it will not be possible to identify your

answers. This study was approved by the Ethics Committee for Social, Life and Health Sciences of the Polytechnic Institute of Viana do Castelo.

Data will be collected using a questionnaire made available online via *Google Forms,* and the study will be divulged to citizens living in continental Portugal aged 18 or over. Data will be collected after participants have given their informed consent to take part in the study. No personal data that would allow the respondents to be identified will be collected, only some sociodemographic data for statistical purposes and answers relevant to the topic in question. The data collected will be stored in a database on the personal computer of the student carrying out the study, protected by a password, to which only the student responsible for the study will have access, to guarantee the security of the data collected, avoiding their loss and reinforcing compliance with ethical and deontological principles. The results will remain anonymous and will be treated with the utmost confidentiality. The data will be used for scientific research purposes only.

If you find anything incorrect, have any questions or would like more information, please contact: Micaela Rodrigues, micaelarodrigues@ipvc.pt or Teresa Mateus, tlmateus@esa.ipvc.pt.

2. QUESTIONNAIRE (as presented in the *Google Forms*)

Please read the following information carefully. If you think something is incorrect or unclear, do not hesitate to ask for more information.

I confirm that I’m over 18:

- Yes
- No

After reading the introductory text, I declare that I have read and understood the information provided to me. I have been guaranteed the possibility of refusing to take part in this study at any time without any consequences. The confidentiality and anonymity of the data collected was guaranteed. I was informed about the period of data preservation. And I was informed that the data would only be used for scientific research purposes.

- Yes
- No

Therefore, I agree to take part in this study and allow the use of the data that I voluntarily provide, trusting that it will only be used for scientific purposes and the guarantees of confidentiality and anonymity given to me by the researcher:

- Yes
- No

2.1 Sociodemographic data

Here you will have to answer the questions with your sociodemographic data. These will not be disclosed, they will only be used for statistical purposes.

1. Gender

- ☐ Female
- ☐ Male
- ☐ Non-binary
- ☐ I rather not to say
- ☐ Other _________

2. Age

____

3. Residence - Municipality

____

4. Residence - Parish

____

5. Education (academic qualification):

- ☐ Illiterate
- ☐ 1st cycle - Infant (4th grade)
- ☐ 2nd cycle - Junior (6th grade)
- ☐ 3rd cycle - 9th grade
- ☐ High school - 12th grade
- ☐ Bachelor/Licentiate
- ☐ Master's degree
- ☐ PhD

6. Job/Occupation

If you are retired or unemployed, please refer your last job or current occupation:

____

2.2 Feelings, attitude and perception about the European hedgehog

1. When you think about these animals, you mostly feel:

	1	2	3	4	5	6	
Just negative feelings							All positive feelings

For you a hedgehog is …. (indicate three words)

____

2. In an eventual encounter with a hedgehog in trouble/danger/injured, what would you do?

- ☐ Seek help
- ☐ Help myself
- ☐ Ignore the situation
- ☐ Try to scare it away
- ☐ Try to kill it

3. In your opinion, do humans have an impact on the mortality of these animals?

	1	2	3	4	5	6	
Very unimportant							Very important

4. What should be done about these animals regarding conservation/management measures?

- ☐ Protect
- ☐ Control the numbers
- ☐ Eliminate

5. Regarding the impact of hedgehogs on agriculture, what is your perception:

	1	2	3	4	5	6	
Very negative							Very positive

2.3 Knowledge about the European hedgehog

1. These mammal populations are…

- Declining
- Stable
- Increasing

2. One can tell the sex of the animal through:

- The distance between the anus and sexual organs
- The presence of testicles in males
- By the size of the animal
- I don’t know

3. In the wild, what’s the average life expectancy of the European hedgehog:

- 3 years
- 5 years
- 10 years
- I don’t know

4.Select the main cause of death of these animals in Portugal?

- Hit by a car
- Sickness
- Starvation
- Poisoning
- Traps
- Body temperature lower than normal (hypothermia)
- I don’t know

5. In your opinion, is it legal to keep European Hedgehogs as pets/in captivity?

- Yes
- No
- I don’t know

6. The European Hedgehog exists in:

- Almost all of Europe
- Almost all of Europe and New Zealand
- All continents
- I don’t know

7. These animals prefer to live in…

- Urban areas (areas with a big population that live and work there)
- Suburban areas (mainly residential areas with more population than rural areas)
- Rural areas (villages and agricultural land)

8. The impact of this species in agriculture is:

	1	2	3	4	5	6	
Very negative							Very positive

9. This species is a…

- Diurnal animal (active during the day)
- Nocturnal animal (active during the night)
- I don't know

10. These animals go into a deep sleep during a certain period of the year. This occurs during the:

- Winter (hibernation)
- Summer (aestivation)
- I don't know

11. The animals are:

- Carnivores (feed on meat, whether it's live or dead animals)
- Omnivores (feed on meat and plants)
- Herbivores (feed on plants)
- Insectivores (feed on insects)
- I don't know

12. Which of the following options represents the largest part of the species' diet:

- Fruits
- Insects, slugs and worms
- Birds
- I don't know

13. Their spines are used for:

- Protection and to cushion falls

- Mating rituals
- Carrying food
- I don’t know

14. When threatened, these animals…

- Tend to be aggressive (bite, scratch, etc.)
- Protect themselves by curling up into a ball
- I don’t know what they do

15. These animals are:

- Solitary
- Live in small communities
- Live in large communities
- I don’t know how they live

16. The most important senses for hedgehogs are:

- Sight and smell
- Smell and hearing
- Sight and hearing
- I don’t know

17. Indicate how many litters a year a female European Hedgehog can have:

- One
- Two
- Three
- I don’t know

18. Who takes care of the cubs?

- The mother
- The father
- The mother and the father
- I don’t know

19. Do hedgehogs have predators?

- Yes
- No
- I don’t know

If you answered "Yes" to the previous question, say which ones:

____

20. Hedgehogs can transmit diseases…

- ☐ To humans
- ☐ To other animals
- ☐ To humans and other animals
- ☐ Hedgehogs do not transmit diseases to humans nor other animals
- ☐ I don't know

If you consider that these animals can transmit diseases to humans, list at least two:

____

2.4 Self-evaluation and past experience

1. How would you rate your knowledge of the biology (morphology, behaviour, reproduction, nutrition, etc.) of the European Hedgehog?

	1	2	3	4	5	6	
Very poor							Very good

2. Have you ever encountered/seen a European hedgehog?

- ☐ Yes, on TV/ magazine/ book/ online
- ☐ Yes, on a biological park or rescue center
- ☐ Yes, on the street/ the countryside/ home
- ☐ No, I have never seen
- ☐ I don't remember if I ever saw
