# Supplementary material for: Prickly Connections: Sociodemographic Factors Shaping Attitudes, Perception and Biological Knowledge about the European Hedgehog
Source: Animals (Basel). 2023 Nov 22;13(23):3610. doi: 10.3390/ani13233610 (PMC10705511; doi:10.3390/ani13233610)
Supplement: Supplementary file 1 [file animals-13-03610-s001.zip › TableS1.pdf]

Supplementary Table for "Prickly connections: sociodemographic factors shaping attitudes, perception and biological knowledge about hedgehog" by Ribeiro et al. 2023.

Table S1. Details about the data collected. Variables used for the statistical analyses and their attributes.

Variables	Type	Attributes
**Dependent**		
**Attitude**<br>Hedgehog in difficulty	categorical	1 – seek help; 2 – help (myself); 3 - ignore; 4 – scare it; 5 - kill
**Feelings**<br>Feelings associated	categorical	1 (all negative) to 6 (all positive)
**Perception**<br>Human impact on mortality	categorical	1 (very unimportant) to 6 (very important)
**Perception**<br>Management/conservation measures	categorical	1 - protect; 2 - control; 3 - eliminate
**Perception**<br>Species impact on agriculture	categorical	1 (very negative) to 6 (very positive)
**Knowledge**<br>State of the population	categorical	1 - declining; 2 - stable; 3 - increasing
**Knowledge**<br>Sexing method	categorical	1 - anogenital distance; 2 – testicles; 3 - size; 4 - I don't know
**Knowledge**<br>Life expectancy (years)	categorical	1 - three; 2 - five; 3 - ten; 4 - I don't know
**Knowledge**<br>Legislation RE captivity	categorical	1 - yes; 2 - no; 3 - I don't know
**Knowledge**<br>Main cause death in PRT	categorical	1- car hit; 2 – sickness; 3- starvation; 4- poisoning; 5 – trap; 6 - hypothermia, 7- I don't know
**Knowledge**<br>Geographic distribution	categorical	1 - Europe; 2 - Europe and New Zealand; 3 - All continents; 4 - I don't know
**Knowledge**<br>Habitat preference	categorical	1 - urban areas; 2 - suburban areas; 3 - rural areas
**Knowledge**<br>Period of activity	categorical	1 - diurnal; 2 - nocturnal; 3 - I don't know
**Knowledge**<br>Period of life in deep sleep	categorical	1 - winter; 2 - summer; I don't know
**Knowledge**<br>Type of diet	categorical	1 - carnivore; 2 - omnivore; 3 - herbivore; 4 - insectivore; 5 - I don't know
**Knowledge**<br>Most abundant items in diet	categorical	1 - fruit; 2 - insects, slugs and worms; 3 - birds; 4 - I don't know
**Knowledge**<br>Spine's function	categorical	1 - protection; 2 - mating; 3- carry food; 4 - I don't know
**Knowledge**<br>Defensive behaviour	categorical	1 - aggression; 2 - curling up; 3 - I don't know
**Knowledge**<br>Sociality features	categorical	1 - solitary; 2 - small communities; 3 – large communities; 4 - I don't know
**Knowledge**<br>Most important senses	categorical	1 - sight and smell; 2 - smell and hearing; 3 - sight and hearing; 4 - I don't know

**Knowledge**<br>Litters per year	categorical	1 - one; 2 - two; 3 - three; 4 - I don't know
**Knowledge**<br>Cubs' caretaker	categorical	1 - mother; 2 - father; 3 - both parents; 4 - I don't know
**Knowledge**<br>Target of predation	categorical	1 - yes; 2 - no; 3 - I don't know
**Knowledge**<br>Disease transmission to	categorical	1 - humans; 2 - other animals; 3 - humans and animals; 4 - don't transmit; 5 - I don't know
**Explanatory**		
Gender	categorical	1 - female; 2 - male; 3 - non-binary
Age	continuous	18 to 93
Typology of parish of residency§	categorical	1 – area predominantly urban (APU); 2 - area moderately urban (AMU); 3 – area predominantly rural (APR)
Academic qualiffication	categorical	1 - illiterate; 2 - 1st cycle – (4th grade); 3 - 2nd cycle (6th grade); 4 - 3rd cycle (9th grade); 5 - High school (12th grade); 6 - Bachelor/Licentiate; 7 - Master's degree; 8 – doctorate
Profession/occupation¶	categorical	1 - legislative and executive power; 2 - intellectual and scientific activities; 3 - intermediate level; 4 - administration; 5 - personal services, security and sales; 6 - agriculture, fishing and forest; 7 - industry, building and craftspeople; 8 - installation, machines and line operators; 9 - unskilled workers; 10 - student; 11 - unemployed/doesn't work; 12 - retired

§ Portuguese classification of urban areas; ¶ Portuguese classification of professions
